# Immune-evasive gene switch enables regulated delivery of chondroitinase after spinal cord injury

**DOI:** 10.1093/brain/awy158

**Published:** 2018-06-14

**Authors:** Emily R Burnside, Fred De Winter, Athanasios Didangelos, Nicholas D James, Elena-Cristina Andreica, Hugo Layard-Horsfall, Elizabeth M Muir, Joost Verhaagen, Elizabeth J Bradbury

**Affiliations:** 1 King’s College London, Regeneration Group, The Wolfson Centre for Age-Related Diseases, Institute of Psychiatry, Psychology and Neuroscience (IoPPN), Guy’s Campus, London Bridge, London, SE1 1UL, UK; 2 Netherlands Institute for Neuroscience, Laboratory for Neuroregeneration, 1105 BA Amsterdam, The Netherlands; 3 Department of Physiology, Development and Neuroscience, University of Cambridge, CB2 3EG, UK

**Keywords:** spinal cord injury, gene therapy, chondroitinase, skilled hand function, neuroplasticity

## Abstract

Chondroitinase ABC is a promising preclinical therapy that promotes functional neuroplasticity after CNS injury by degrading extracellular matrix inhibitors. Efficient delivery of chondroitinase ABC to the injured mammalian spinal cord can be achieved by viral vector transgene delivery. This approach dramatically modulates injury pathology and restores sensorimotor functions. However, clinical development of this therapy is limited by a lack of ability to exert control over chondroitinase gene expression. Prior experimental gene regulation platforms are likely to be incompatible with the non-resolving adaptive immune response known to occur following spinal cord injury. Therefore, here we apply a novel immune-evasive dual vector system, in which the chondroitinase gene is under a doxycycline inducible regulatory switch, utilizing a chimeric transactivator designed to evade T cell recognition. Using this novel vector system, we demonstrate tight temporal control of chondroitinase ABC gene expression, effectively removing treatment upon removal of doxycycline. This enables a comparison of short and long-term gene therapy paradigms in the treatment of clinically-relevant cervical level contusion injuries in adult rats. We reveal that transient treatment (2.5 weeks) is sufficient to promote improvement in sensory axon conduction and ladder walking performance. However, in tasks requiring skilled reaching and grasping, only long term treatment (8 weeks) leads to significantly improved function, with rats able to accurately grasp and retrieve sugar pellets. The late emergence of skilled hand function indicates enhanced neuroplasticity and connectivity and correlates with increased density of vGlut1+ innervation in spinal cord grey matter, particularly in lamina III–IV above and below the injury. Thus, our novel gene therapy system provides an experimental tool to study temporal effects of extracellular matrix digestion as well as an encouraging step towards generating a safer chondroitinase gene therapy strategy, longer term administration of which increases neuroplasticity and recovery of descending motor control. This preclinical study could have a significant impact for tetraplegic individuals, for whom recovery of hand function is an important determinant of independence, and supports the ongoing development of chondroitinase gene therapy towards clinical application for the treatment of spinal cord injury.

## Teaser

Chondroitinase is a promising preclinical therapy for restoring function after spinal cord injury. Burnside *et al.* engineer an immune-evasive vector system that allows delivery of chondroitinase under the control of an on/off switch to regulate expression. Switching on chondroitinase helps spinal-injured rats to recover reaching and grasping movements.

## Introduction

Spinal cord injury results in permanent disruption to nervous system function, for which there is no current regenerative or pathology-modifying treatment (Ramer *et al.*, 2014). Following CNS injury, reactive glia synthesize and secrete chondroitin sulfate proteoglycans (CSPGs) into the extracellular matrix (McKeon *et al.*, 1991). CSPGs have one or more covalently attached chondroitin sulfate glycosaminoglycan (CS-GAG) side-chains, which are known to inhibit neuronal extension and plasticity. Removal of CS-GAGs by the chondroitinase ABC (ChABC) enzyme reverses neurite growth arrest *in vitro* (Smith-Thomas *et al.*, 1994), enhances CNS axonal regeneration and neuroplasticity *in vivo* (Moon *et al.*, 2001; Alilain *et al.*, 2011) and promotes functional recovery following spinal cord injury in adult rats (Bradbury *et al.*, 2002; Garcia-Alias *et al.*, 2009). This effect has been replicated across multiple laboratories, species and CNS injury models (Bradbury and Carter, 2011), and recently in a canine clinical trial (Hu *et al.*, 2018). A gene therapy method of enzyme delivery, where host cells are themselves transduced to express the *ChABC* gene, circumvents the need for repeated, invasive administration of the enzyme, which has low thermal stability (Lee *et al.*, 2010) and a short half-life that is thought to limit efficacy in severe models of trauma that mimic clinical pathology (Tom *et al.*, 2009). We have previously reported a gene therapy strategy whereby optimization of the prokaryotic *ChABC* gene to render it compatible with translation and secretion from mammalian cells (Muir *et al.*, 2010) and incorporation of this gene into viral vectors (Zhao *et al.*, 2011), leads to high levels of *ChABC* gene expression and active enzyme release *in vivo.* This results in extensive CS-GAG digestion across many segments of the mammalian spinal cord (Bartus *et al.*, 2014). This large scale matrix modification leads to reduced tissue pathology and improved functional recovery following contusion injury to the thoracic (Bartus *et al.*, 2014) and cervical (James *et al.*, 2015) spinal cord. Thus, *ChABC* gene therapy is a promising therapeutic strategy for the treatment of spinal cord injury.

However, uncontrolled gene expression can limit or even reverse therapeutic benefits (Georgievska *et al.*, 2004; Fouad *et al.*, 2013). Therefore, the ability to both administer and remove any treatment has a key advantage over permanent application in the development of clinically feasible strategies. Furthermore, most current clinical applications of gene therapy for treating human disorders are targeted mainly to rare genetic conditions with rapid progressive decline or fatal neurodegenerative diseases (Mendell *et al.*, 2017), where any negative effects of gene delivery, which cannot be switched off, carry less risk in terms of cost-to-benefit analysis (Bender, 2016). Spinal injured individuals, however, represent a higher risk group for irreversible gene therapy since they are a relatively stable population. Following the initial trauma, early surgical and medical interventions and a period of adjustment during which any spontaneous recovery reaches a plateau, the majority of spinal injured individuals remain in a stable condition for the rest of their lives, albeit severely debilitated (Ahuja *et al.*, 2017). Thus, an important safety consideration in the development of gene therapies for spinal cord injury and other long term neurological disorders is the ability to control transgene expression. Effective methods of regulating gene expression in the CNS would also expand the number of conditions that are amenable to gene therapy treatment. While there is no evidence that sustained *ChABC* gene therapy, at least for up to 12 weeks, has any negative effects in rats (Bartus *et al.*, 2014), given its potent effects on neuroplasticity, the ability to switch off the gene represents a significant step towards generating a more clinically feasible treatment. Moreover, it provides an experimental tool to ask how temporal regulation of ChABC delivery influences treatment efficacy. This could provide important insight regarding therapeutic timing since, despite a wealth of positive preclinical findings, little is known as to how timing of ChABC administration could affect functional outcome and whether long term administration would have any additional advantage over acute transient delivery. Doxycycline-regulated Tet-On systems are widely used inducible gene expression platforms that have been optimized for use *in vitro* (Urlinger *et al.*, 2000; Zhou *et al.*, 2006; Loew *et al.*, 2010) and have shown promise in some *in vivo* experiments (Stieger *et al.*, 2009). However, particularly when studies are scaled-up to testing in non-human primates, experiments have shown that an immune response may be generated against the system transactivator, resulting in cytotoxic T cell-mediated removal of cells expressing the therapeutic transgene (Favre *et al.*, 2002; Latta-Mahieu *et al.*, 2002; Ginhoux *et al.*, 2004). This phenomenon was not overcome by replacing activation domains with humanized moieties (Le Guiner *et al.*, 2014)*.* Hence, *in vivo* use, and further translation of transactivator-based systems is compromised by immune recognition and removal. The injured spinal cord has a complex and non-resolving inflammatory profile, comprising both innate and adaptive responders (Fleming *et al.*, 2006; Pruss *et al.*, 2011; reviewed in Ankeny and Popovich, 2009; David and Kroner, 2011) and removal of cells expressing a therapeutic transgene would not only prevent treatment, but could itself confer damaging neuronal or glial loss if these cells are transduced to express the transgene. We therefore sought to use an immune-stealth modification, created by fusing the reverse tetracycline-controlled transactivator with a glycine-alanine repeat (GARrtTA) (Zaldumbide *et al.*, 2010; Hoyng *et al.*, 2014), which is known to enable the Epstein-Barr virus to evade T lymphocyte-mediated removal (Levitskaya *et al.*, 1997; Yin *et al.*, 2003). This enabled the development of a potentially immunologically-inert, doxycycline-inducible, gene delivery system to stably and controllably deliver ChABC to injured tissue within the environment of a traumatic spinal cord injury.

Here we assess the efficacy of a novel dual lentiviral immune-evasive doxycycline-inducible ChABC (dox-i-ChABC) vector system. In this system *ChABC* gene expression is regulated via the widely clinically-available broad-spectrum antibiotic doxycycline (dox). Since the majority of spinal injured individuals are injured at the cervical level (National Spinal Cord Statistical Centre, 2017) and a top priority for improving independence and quality of life is recovery of hand and digit function (Anderson, 2004), we investigate the effect of dox-i-ChABC treatment following a clinically relevant cervical spinal contusion injury model in adult rats. This model closely mimics the pathology and progression of spinal cord injury found clinically and affects functions of the upper limb and hand. We demonstrate effective regulation of ChABC delivery following injury, where doxycycline administration and removal controls *ChABC* gene expression. Using this tool, we switch on ChABC transiently or maintain delivery long term following injury and demonstrate task-specific therapeutic temporal dependence, whereby short-term dox-i-ChABC treatment is sufficient to mediate improvements in a task of sensorimotor ability that correlates with increased ascending sensory transmission and sustained dox-i-ChABC treatment confers additional later benefit in a measure of skilled hand function, associated with anatomical evidence of neuroplasticity of descending motor pathways.

## Materials and methods

### Generation and *in vitro* testing of the immune-evasive dox-i-ChABC system

The mammalian-compatible *ChABC* gene has been described previously (Muir *et al.*, 2010). Briefly, the gene contains codons optimized for expression in mammalian cells, an optimized 5′ Kozak sequence, and a mouse matrix metalloproteinase-2 signal sequence for secretion. It is also modified by removal of specific post-translational *N*-glycosylation sites, which impede passage of the prokaryotic enzyme through the eukaryotic secretory machinery (Muir *et al.*, 2010). This transgene was cloned into a lentiviral transfer vector (pRRLsin-PPth) where gene expression is under control of a tetracycline response element (TRE) comprising eight TetO repeats (p8teto-36ΔI) and a cytomegalovirus minimal promoter (Agha-Mohammadi *et al.*, 2004; Hoyng *et al.*, 2014) (pLV.TRE.ChABC). A second transfer vector was generated in which the immune-evasive transactivator GARrtTA (Zaldumbide *et al.*, 2010) is constitutively expressed from a full-length *PGK1*/PGK promoter (pLV.PGK.GARrtTA).

#### *In vitro* assessment of dox-i-ChABC plasmids

Human embryonic kidney 293T (HEK293T) cells grown in SensiCell™ medium supplemented with 10% foetal calf serum (FCS) were plated into 6-well plates and left overnight to achieve 70% confluency. Cells were then washed twice with Dulbecco’s modified Eagle medium and transfected using Xfect™ (Clontech) in serum free medium for 4 h, with a total of 3 µg DNA consisting of the TRE.ChABC and PGK.GARrtTA plasmids in a ratio of 1:4 and 0.3 µg pAdVAntage™ plasmid (Promega). pAdVAntage™ was added to enhance expression of the *ChABC* gene (Muir *et al.*, 2010). Following transfection, medium was removed and replaced with doxycycline-free medium containing 10% FCS (Clontech). Doxycycline was then added at 1, 10 and 100 ng/ml. Medium and cells were harvested after 48 h and enzyme levels in the medium measured by enzymatic colorimetric assay (Morgan Elson reaction), *n = *5, 3, 3 and 6 for 0, 1, 10 and 100 ng/ml, respectively, where *n = *individual well normalized to the maximum activity in each plate.

#### Dox-i-*ChABC* gene therapy dual lentiviral vector generation

**Figure 1:**
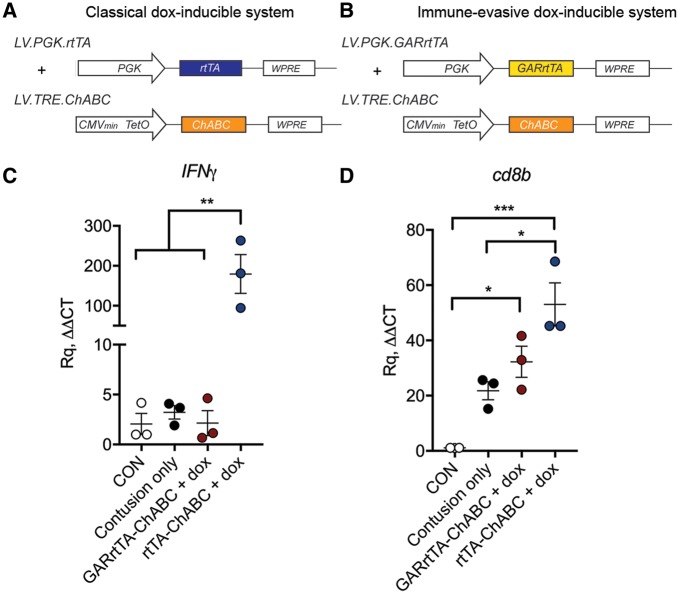
**Doxycycline-inducible dual vector system for regulated delivery of ChABC with immune-evasive gene switch (dox-i-ChABC).** (**A** and **B**) Linear diagram of lentiviral vectors used in a side by side comparison of two dual-vector dox-inducible systems for delivering the ChABC transgene, using either (**A**) a classical transactivator system (rtTA) or (**B**) a chimeric transactivator construct (GARrtTA) designed to evade recognition by T cells. (**C**) Expression of IFNγ in tissue immediately rostral and caudal to the injury epicentre at 2.5 weeks following contusion injury and intraspinal injection with rtTA-ChABC, GARrtTA-ChABC [and doxycycline (dox) delivery] or contusion only, relative to uninjured tissue (CON), revealed a dramatic increase in IFNγ expression in the classical rtTA group in comparison to all other groups [*F*(3,8) = 13.17, *P = *0.0018, one-way ANOVA, Tukey’s *post hoc*]. (**D**) The expression of CD8b was upregulated by contusion injury and further increased in the rtTA group, but not when the ChABC transgene was delivered with the ‘stealth’ GARrtTA gene switch [*F*(3,8) = 18.18, *P = *0.006, one-way ANOVA, Tukey’s *post hoc*], indicating differential immune response to these two regulated delivery systems. The immune-evasive GARrtTA dox-inducible vector (dox-iChABC) was used throughout the remainder of the study.

**Figure 2:**
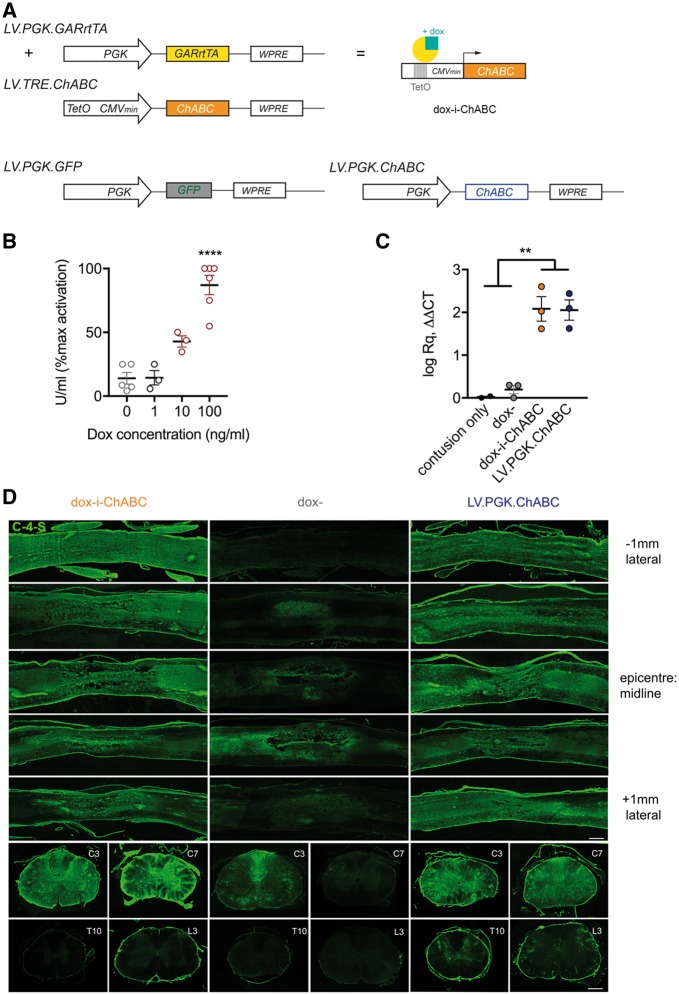
**Dox-i-ChABC enables doxycycline inducible gene expression and secretion of active ChABC *in vitro* and *in vivo*.** (**A**) Linear diagram of lentiviral vectors used in the study and depiction of gene expression induced by doxycycline (dox) interaction with TRE and GARrtTA constructs. (**B**) *In vitro* co-transfection of dox-i-ChABC plasmids confer dox-sensitive expression of active ChABC enzyme in HEK cells [*F*(3,13) = 32.45, *P = *0.0001 one-way ANOVA]. Medium from co-transfected cells had some low basal ChABC activity (14% ± 4.5 U/ml), which was not increased by 1 ng/ml doxycycline administration. Application of 10 ng/ml doxycycline initiated the induction of *ChABC* gene expression (43% ± 4.4 U/ml), relative to maximal activity achieved by application of 100 ng/ml doxycycline (87% ± 7.6 U/ml). (**C**) *In vivo* expression of the *ChABC* gene is induced by dox-i-ChABC treatment to the same magnitude as LV.PGK.ChABC, measured by qPCR on extracted RNA from spinal cord tissue at 2.5 weeks following contusion injury and intraspinal injection [*F*(3,7) = 27.13, *P = *0.0003, one-way ANOVA, Tukey’s correction for multiple comparisons]. (**D**) Translation and secretion of active enzyme was assessed by immunostaining for C-4-S in sagittal spinal cord sections through the C5/6 lesion epicentre (*top five rows*) and at C3, C7, T10 and L3 at 2.5 weeks following contusion and intraspinal injection. Dox-i-ChABC (*left*) induced intense C-4-S expression throughout the lesioned tissue, to a comparable magnitude to LV.PGK.ChABC (*right*). This extended to spinal levels rostral (C3) and caudal (C7) to the lesion. At 2.5 weeks, little C-4-S is observed in thoracic (T10) or lumbar (L3) spinal cord, with a small amount of basal ‘leaky’ expression detectable without doxycycline (*middle*), localized around the injection sites. Scale bar = 0.5 mm.

Lentiviral vectors (second generation) were generated as described previously (Naldini *et al.*, 1996; Hendriks *et al.*, 2007). In short, lentiviral stocks were produced by co-transfection of 3 plasmids; VSV-G envelope protein vector (pMD.G.2), the viral core packaging vector (pCMVdeltaR8.7.4) and the transfer vector plasmid (pLV.TRE.ChABC or pLV.PGK.GARrtTA) into HEK293T cells. Forty-eight hours after transfection, lentiviral particles were harvested from the medium (Iscove’s modified Dulbecco’s medium supplemented with 2% FCS and 2 mM GlutaMAX, Sigma Aldrich) by ultracentrifugation and the viral particle-containing pellet was dissolved in phosphate-buffered saline (PBS, pH 7.4). Lentiviruses were aliquoted and stored at − 80°C until further use. Lentiviral stocks were titred by infecting HEK293T cells in serial dilutions followed by a quantitative PCR (qPCR) (using SYBR® Green, Applied Biosystems) for the Woodchuck hepatitis virus post-transcriptional regulatory element (WPRE) to determine the number of inserted viral genomic copies (GC) into the host cell genomic DNA. WPRE qPCR primer sequences used forward: 5′-TTCCCGTATGGCTTTCATTT-3′ and reverse: 5′ GAGACAGCAACCAGGATTTA-3′. GAPDH qPCR reference primer sequences used forward: 5′-CATGGCCTCCAAGGAGTAAG-3′ and reverse: 5′-AGGGGTCTACATGGCAACTG-3′. LV.TRE.ChABC and LV.PGK.GARrtTA were mixed and used in a 1:1 ratio to result in a working titre of 10^10^ GC/ml for each vector. Other lentiviral vectors used in this study were: LV.PGK.ChABC (Bartus *et al.*, 2014) (positive control), LV.PGK.GFP (negative control) and PGK.rtTA and LV.TRE.ChABC in a 1:1 ratio (control for immune-evasive properties of GAR), all at a titre of 10^10^ GC/ml. Schematic diagrams of vectors used in this study are shown in Figs 1A and 2A.

### Experimental design

#### Animals

Eighty-five adult female Lister Hooded rats (240–250 g; Charles River) were used in these studies. Rats were housed under a 12-h light/dark cycle with *ad libitum* access to food and water. All procedures were performed in accordance with the United Kingdom Animals (Surgical Procedures) Act 1986, approved by the Animal Welfare and Ethical Review Body (AWERB) of King’s College London and conducted under Home Office Project License 70/8032. Methods and results are written in accordance with the ARRIVE guidelines for publishing *in vivo* research.

### Study design

#### Assessing inducible gene expression with dox-i-ChABC

To assess whether ChABC expression could be induced by doxycycline administration and to compare the novel dox-i-ChABC system with our previous constitutive *ChABC* gene therapy system, negative control rats received a contusion injury and either no treatment (contusion only; *n = *2 for qPCR), or dox-i-ChABC vector injection without doxycycline administration (dox–; *n = *3 for qPCR, *n = *2 for histology). Positive control rats received LV.PGK.ChABC vector injection (*n = *3 for qPCR, *n = *2 for histology). Dox-i-ChABC treated rats received dox-i-ChABC dual vector injections and were administered doxycycline for 2.5 weeks (*n = *3 for qPCR, *n = *2 for histology). All tissue was processed at 2.5 weeks for evidence of *ChABC* gene expression at the mRNA level and for histological evidence of digested CS-GAGs, see below. Sample sizes for biochemical quantitation are consistent with prior data (Didangelos *et al.*, 2014), and generated using [type 1 error threshold (α) ≤ 0.05 and power (1 − β) ≥ 0.80].

#### Assessing validity of on/off switch and efficacy of long- versus short-term dox-i-ChABC after spinal contusion injury

**Figure 3:**
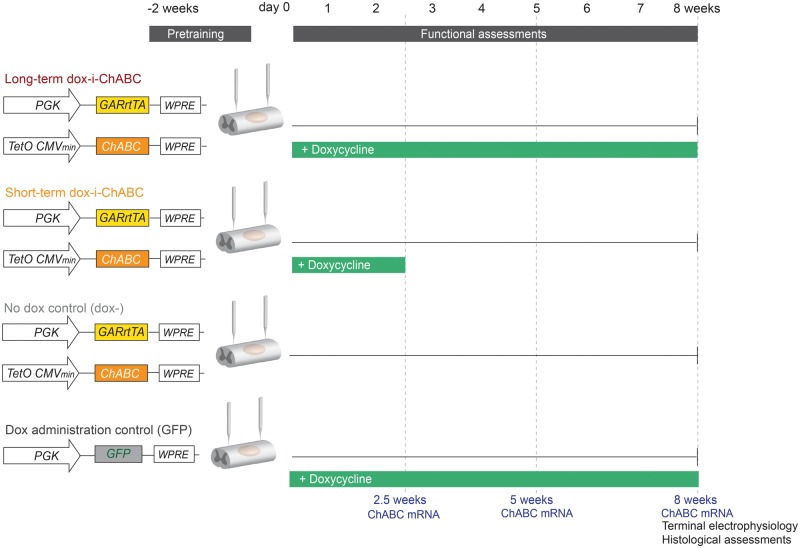
**Dox-i-ChABC study design.** To validate the on/off switch over time and assess functional efficacy of long term versus short term dox-i-ChABC, animals were randomly assigned to four treatment groups following pretraining in behavioural tasks and midline cervical 225 kdyn contusion injury (dox administration is in green): (*from top to bottom*) Dox-i-ChABC vectors with doxycycline administration sustained for the 8 week duration of the study (long-term dox-i-ChABC); Dox-i-ChABC vectors with doxycycline administration for the first 2.5 weeks (short-term dox-i-ChABC); Dox-i-ChABC vectors without doxycycline administration (dox−); and GFP vector injection with doxycycline administration throughout (GFP), to control for broad-spectrum antibiotic treatment. Additional animals were also included to assess *ChABC* gene expression at different time points throughout the experiment (dotted lines). Terminal electrophysiology and histological assessments were performed after the final behavioural time point.

A long-term study was designed (Fig. 3) in which there were four treatment groups following *in vivo* spinal contusion injury: dox-i-ChABC dual vector injection and doxycycline administration for 8 weeks (long-term dox-i-ChABC), dox-i-ChABC dual vector injection and doxycycline administration for 2.5 weeks (short-term dox-i-ChABC), dox-i-ChABC dual vector injection without doxycycline administration (dox−) and GFP vector injection and doxycycline administration for 8 weeks (GFP). *ChABC* gene expression was assessed by qPCR at 2.5 weeks, 5 and 8 weeks (*n = *3 per group per time point). Animals were assessed weekly in behavioural tasks (*n = *7, 7, 7 and 5 for long-term dox-i-ChABC, short-term dox-i-ChABC, dox− and GFP, respectively). The present study had no *a priori* expectation as to effect size, as dox-i-*ChABC* gene therapy has not been used before in this context. To select sample size, G*Power 3.17 was used to suggest a sample size of *n = *7 per group would be sufficient to detect an effect size less than that observed in a previous cervical contusion study using LV.PGK.ChABC in the horizontal ladder task (James *et al.*, 2015), accounting for differences in group number and number of weeks of repeated assessments [type 1 error threshold (α) ≤ 0.05 and power (1 − β) ≥ 0.80]. At the end of behavioural assessments, *n = *4 per group underwent terminal electrophysiological assessment of sensory axon conduction. This sample size is consistent with previous studies (James *et al.*, 2011). Tissue from animals was subsequently taken for histological analyses (methodology detailed below).

#### Blinding and randomization

For all animals used in behavioural studies the experimenter performing analysis was blind to the treatment groups. During surgical procedures this was ensured by the experimenter injecting the vectors being unaware of animal identification number. Animals across the three groups receiving doxycycline administration were randomized into cages, where an experimenter unaware of treatment group randomly assigned caging. The dox− group were, by necessity, in the same cages initially, since they were the only group not to receive doxycycline chow; however, at the 2.5 week time point when doxycycline was removed from the short-term dox-i-ChABC group, all animals were again randomized. Thus, blinding was maintained as far as possible during data collection and all behaviour was video recorded and analysed with the experimenter blind to treatment. Electrophysiological assessments were also performed with the experimenter blind to treatment.

### Surgical procedures and treatment

Adult female Lister Hooded rats (*n = *70) were given perioperative analgesia (carprofen, Carprieve™, 5 mg/kg, administered subcutaneously) and anaesthetized (ketamine, 60 mg/kg, and medetomidine, 0.25 mg/kg, administered intraperitoneally). Skin was shaved and cleansed with sequential chlorohexidine and iodine swabs. Overlying muscle was retracted and a dorsal laminectomy of C6 vertebral process and half of C5 vertebral process was performed. Periosteum was removed. Rats then received a midline 225 kdyn spinal cord contusion injury at level C5/6 using an Infinite Horizon impactor and 3-mm diameter rounded impact tip (Precision Systems Instrumentation). Immediately following injury, rats underwent midline intraspinal injection of viral vectors at two sites, 1 mm rostral and 1 mm caudal to the injury, using a fine glass pulled-pipette and microdrive pump (NanoLiter 2010 Injector/Micro 4 Controller, World Precision Instruments). The pipette was lowered 1.5 mm in the dorsoventral axis then retracted 0.5 mm and vectors were injected at a rate of 200 nl/min. The pipette was left in place for a further 2 min to ensure vector diffusion. Viral vectors were injected at a volume of 1 µl per site (LV.PGK.GARrtTA and LV.TRE.ChABC mixed in a 1:1 ratio; *n = *55) or 0.5 µl (LV.PGK.ChABC, *n = *5, and LV.PGK.GFP, *n = *8). Following intraspinal injection, overlying musculature was sutured in layers, skin was closed and anaesthesia was reversed (atipamezole hydrochloride, 1 mg/kg, administered subcutaneously). Body temperature was maintained at 37°C using a self-regulating heating mat throughout and animals recovered for at least 1 h in an incubator (water base thermostat 32°C, Thermocare) and were then returned to home cages placed partially on heated mats overnight (∼35°C). For animals receiving doxycycline, this was administered at a dose of ∼80 mg/day via custom chow (Envigo). In the acute postoperative period following surgery, extensive welfare checks were carried out, including provision of accessible chow and hydration gel in all cages. During this period animals were housed in soft bedding and absorbent-lined cages with less sawdust, to maximize ability to locomote, before returning to standard husbandry conditions.

#### Assessing immune-evasive properties of the ‘stealth’ gene switch

To assess the immune-evasive properties of dox-i-ChABC, an additional cohort of animals (*n = *12) was prepared for comparisons of T cell-related gene expression in animals receiving the dox-i-ChABC dual vector system compared to animals with the same dual vector system without the immune-evasive (GAR) component. Rats received a 225 kdyn contusion injury at the C5/6 level, as described above, followed by intraspinal injections 1 mm rostral and caudal to the lesion with 1 µl of LV.PGK.rtTA and LV.TRE.ChABC (in a 1:1 ratio, working titre 10^10^ GC/ml) (*n = *3) or LV.PGK.GARrtTA and LV.TRE.ChABC (in a 1:1 ratio, working titre 10^10^ GC/ml) (*n = *3), or received no intraspinal injection (contusion only, *n = *3). Animals injected with inducible vectors received daily doxycycline (80 mg) for 2.5 weeks and were then deeply anaesthetized with sodium pentobarbital (Euthatal®, 80 mg/kg, administered intraperitoneally) and transcardially perfused with 50 ml PBS supplemented with EDTA (12.5 mM). Two sections of spinal cord rostral and caudal to the 8 mm injury epicentre were rapidly dissected and frozen on dry ice and stored at −80°C until processing. Tissue was thawed on ice and homogenized in 600 μl TRIzol® reagent (Thermo Fisher Scientific). An aqueous (RNA-containing) phase was generated using 1:10 bromo-chloro-propane, mixed 1:1 with 70% isopropanol and centrifuged at 12 000 rpm to precipitate RNA. RNA samples (3000 ng) were pooled in a 1:1 ratio from the rostral and caudal samples and converted into cDNA using the high capacity RNA-to-cDNA™ kit (Applied Biosystems). Thirty nanograms of RNA per reaction was quantified using prevalidated Taq Assays for interferon gamma (IFNγ) and CD8b (Thermo Fisher). *GAPDH* served as the housekeeping gene, using prevalidated *GAPDH* primers (Roche). RT-qPCR (TaqMan™ assay) was performed using an automated cycler (Roche LightCycler® 480 II) and mRNA expressed as ΔΔCt relative to control tissue from uninjured rats (*n = *3).

### Behavioural functional assessments

#### Horizontal ladder

Prior to injury, animals were trained 15 min daily for 2 weeks to cross a 1-m horizontal ladder with unevenly spaced rungs and baseline values were obtained. Across the course of three video-recorded runs along the ladder, total forelimb and hindlimb steps were quantified and rated as successful or incorrect. Steps were rated incorrect if the paw slipped from the rung (partially or fully) or were non-weight bearing. This was expressed as a percentage of total steps. Following injury, animals were assessed weekly on this task. They were assessed in chronological numerical order, which was independent and unrelated to treatment group, during the morning.

#### Single pellet reaching: Whishaw window

Prior to injury, animals were trained 15 min daily for 2 weeks to reach for sucrose pellets (Test Diet, Sandown Scientific), with their dominant paw, through a window in a Plexiglas® box. Pellets were placed in an indented well, retrieval from which requires grasping and paw supination rather than dragging of the pellet towards the mouth. During baseline and testing, rats were offered 10 practice pellets and then 20 ‘trial’ pellets which were scored from the dominant paw. ‘Hits’ (trials resulting in first-time pellet acquisition) were recorded and expressed as a percentage of ‘trials’ (Whishaw *et al.*, 2008). Rats were not trained to return to the back of the testing arena each time as we wished to assess grasping ability independently of any general locomotor deficit confound. For inclusion in assessment all rats achieved >65% pellet retrieval success at baseline. Sessions were video recorded at ≥60 frames per second and rats were not food-restricted prior to testing. Following injury, animals were assessed weekly. They were assessed in chronological numerical order, which is independent and unrelated to treatment group, during the morning. Rats were given any sugar pellets they failed to retrieve after the task.

#### Manual 2D kinematic analysis of reaching and grasping

A representative sample of rats were taken from the short and long-term dox-i-ChABC treatment groups (*n = *3 from each group, the worst, median and best performers from prior analysis). Videos from the 8-week time point were split into single frames and the middle proximal phalange position recorded in sequential frames using a Manual Tracker plugin (Fiji). Traces were smoothed via a single interpolation for graphical representation. The *y*-coordinate, representing window opening, was used to select *x–y* information as to paw location during ‘grasp phase’. This is defined as having been initiated when the paw passes through the window. Heat maps of relative paw position during this time were generated using the NumPy and Matplotlib/Pyplot histogram2d function in Python 3.6. From these traces and known frame length, the average length of time the rat took to successfully grasp the pellet was also calculated from onset of first passage of the paw through the window opening to successful retrieval of that pellet.

### Analysis of *ChABC* gene expression by quantitative PCR

Animals were deeply anaesthetized with sodium pentobarbital (Euthatal®, 80 mg/kg, administered intraperitoneally) and transcardially perfused with 50 ml PBS supplemented with EDTA (12.5 mM). A section of spinal cord spanning injury epicentre and injection site (∼8 mm) was rapidly dissected and frozen on dry ice and stored at −80°C until processing. Tissue was thawed on ice and homogenized in 700 μl TRIzol® reagent (Thermo Fisher Scientific). An aqueous (RNA-containing) phase was generated using 1:10 bromo-chloro-propane, mixed 1:1 with 70% isopropanol and centrifuged at 12 000 rpm to precipitate RNA. RNA was converted to cDNA using the high capacity RNA-to-cDNA™ kit (Applied Biosystems). Thirty nanograms of RNA per reaction was quantified using primers generated against the modified *ChABC* gene at a concentration of 100 nM (Forward: 5′-AGAGCCGTAGGCGTCTCTCT-3′. Reverse: 5′-AGCGTTGAGGGTCATCTCTC-3′). *GAPDH* served as the housekeeping gene, using prevalidated *GAPDH* primers (Roche). RT-qPCR (TaqMan™ assay) was performed using an automated cycler (Roche LightCycler® 480 II). The log fold change in mRNA expression of *ChABC* was calculated from ΔΔCt values.

### Immunohistochemistry

Animals were deeply anaesthetized with sodium pentobarbital (Euthatal®, 80 mg/kg, administered intraperitoneally) and transcardially perfused with PBS followed by 4% paraformaldehyde in 0.1 M phosphate buffer. Tissue spanning the lesion site (C5/6), plus C3/4, C7/C8, T10 and lumbar spinal cord was dissected and post-fixed overnight, cryoprotected in 30% sucrose, embedded and frozen in O.C.T. compound and then cut into serial 20 µm transverse or sagittal sections using a cryostat. To detect ChABC enzyme activity, immunohistochemistry for the terminal non-reducing C-4-S product, indicative of ChABC digestion of CS-GAGs, was performed (Barritt *et al.*, 2006). Briefly, mouse monoclonal anti-C-4-S (1:5000; MP Biomedicals) immunohistochemistry was amplified using tyramide signal amplification and detected using extra-avidin FITC (1:500). Images were acquired at ×20 using a Zeiss LSM710 confocal microscope. To assess excitatory glutamatergic innervation, transverse C4/5 and C7/8 sections were immunostained using a rabbit anti-vGlut1 antibody (1:500, Synaptic Systems) and Alexa Fluor® 488 secondary antibody (1:1000, Life Technologies). These were imaged and analysed as described below. A further series of C4/5 and C7/8 sections from the long-term dox-i-ChABC and short-term dox-i-ChABC groups were double stained for vGlut1, as above, and a neuronal marker (mouse monoclonal biotinylated anti-NeuN 1:500 Millipore followed by extra avidin TRITC 1:500, Sigma) for further examination of vGlut1 expression in these two treatment groups. These images were acquired on a Zeiss LSM10 confocal microscope at ×40. To detect serotoninergic fibres, transverse T2 sections were immunostained using rabbit polyclonal anti-5HT (1:15 000; ImmunoStar) and Alexa Fluor® 488 secondary antibody (1:1000, Life Technologies) and imaged at ×20 using a Zeiss LSM710 confocal microscope.

### VGlut1 analysis

Transverse sections of C4/5 spinal cord were imaged at ×10 on a Zeiss LSM10 confocal microscope and transverse sections of C7/8 spinal cord were imaged at ×10 on a Ziess Z1 widefield fluorescence microscope. Images were converted to 8-bit, rigid orientation-registered to a spinal cord template section corresponding to the appropriate spinal level by feature mark-up, and batch processed using custom macros for threshold and regional-specific mask application for spinal laminas I–II, III–V and VI–X (Fiji). A pixel value threshold was assigned as representative of positive immunostaining and maintained across all analysis for C4/5 analysis and again for C7/8 analysis. Pixels above this threshold were summed and averaged for laminas I–II, III–V and VI–X, across three sections per animal in four to five animals per treatment group alongside three uninjured controls. Averaged threshold grey matter data per group were then converted to heat maps to display density of vGlut1+ staining using NumPy and Matplotlib/Pyplot histogram2d function in Python 3.6.

#### 5HT analysis

Three transverse sections per animal were imaged at ×20 on a Zeiss LSM 710 confocal microscope and regions of interest applied over an area comprising the intermediolateral columns and ventral horns. These regions were assigned a pixel threshold representative of positive immunostaining that was maintained across all analysis, whereby pixels above this threshold were summed and averaged across each animal (*n = *3) in each experimental group.

### Electrophysiology

At 8 weeks post-injury, terminal electrophysiological experiments were performed to assess the conduction properties of dorsal column sensory axons through the injury site (James *et al.*, 2015). Rats were deeply anaesthetized with urethane (1.25 g/kg, administered intraperitoneally) and body temperature was maintained close to 37°C using a self-regulating heated mat. A lumbar cord laminectomy was performed to expose dorsal roots from L3–S1, followed by a separate cervical exposure of the injury site to enable placement of stimulating electrodes rostral and caudal to the injury. Following both spinal cord exposures, the dura was removed and mineral oil applied over CNS tissue. Filaments of dorsal roots L3–S1 on the left and right hand side, were teased using sharp forceps and individually mounted on silver wire recording electrodes in order to record ∼10 single-unit antidromic responses to a stimulus of 0.2-ms duration square wave pulses at a frequency of 1 Hz caudal to the lesion. Increasing stimulus amplitude (0–800 μA) recruits sequentially more all-or-none unit responses, whereby all action potentials in the filament were typically present by 400 μA. The stimulating electrode was then switched rostral to the lesion and the single units remaining quantified and expressed as a percentage of those recruited when stimulating caudal to the lesion.

### Statistical analysis

Numerical values are reported as mean ± standard error of the mean (SEM). The experimental unit (*n*) is the rat. Parametric statistical analyses were performed using IBM SPSS Statistics 24 or GraphPad Prism 7. Behavioural data were analysed by two-way repeated measures analysis of covariance (RM-ANCOVA), where baseline performance was a covariate. Assumptions of ANCOVA, including normality, homogeneity of error variances and sphericity were tested and satisfied. Histological and electrophysiological data were analysed by one-way ANOVA (analysis of variance) or *t*-test. All statistics and *post hoc* tests are stated in the text and correction for multiple comparisons performed where appropriate. Error bars are SEM unless stated.

## Results

### Addition of GAR peptide confers immune evasive properties to the inducible gene switch

Expression of IFNγ (known to be predominantly produced by T cells) and CD8b (a CD8+ T cell marker) was examined in tissue immediately rostral and caudal to the epicentre (the injury penumbra rather than epicentre was examined because of the mass influx of immune cells at the epicentre, which would likely mask any vector-related changes) in animals receiving intraspinal injections of dox-inducible vectors, which were designed with either a classical transactivator (rtTA) to activate gene expression upon doxycycline administration (Fig. 1A), or an identical vector design except for the addition of the GAR peptide (GARrtTA), to create a ‘stealth’ gene switch designed to evade the immune system (Fig. 1B). At 2.5 weeks following injury and daily doxycycline treatment, a dramatic upregulation of IFNγ was observed in the classical reverse tetracycline controlled transactivator (rtTA) group in comparison with all other groups [*F*(3,8) = 13.17, *P = *0.0018, one-way ANOVA, Tukey’s *post hoc*; Fig 1C]. Moreover, while CD8b was found to be increased by contusion only, it was further increased in the classical rtTA group, but not the GARrtTA group [*F*(3,8) = 18.18, *P = *0.006, one-way ANOVA, Tukey’s *post hoc*; Fig. 1D]. These data indicate a differential immune response between the classical rtTA and GARrTA dox-inducible ChABC vector systems, with no IFNγ response and no enhanced CD8b response in the GARrTA group. Thus, we provide an *in vivo* indication that our GAR peptide confers immune-evasive properties to the inducible vector system. In all subsequent studies, we used the GARrtTA stealth system (termed dox-i-ChABC).

### *In vitro* co-transfection of PGK.GARrTA and TRE.ChABC plasmids confers dox-sensitive ChABC enzyme activity

We next sought to assess whether the PGK.GARrtTA and TRE.ChABC constructs could be used to induce synthesis of active ChABC enzyme in the presence of doxycycline *in vitro.* HEK cells transfected with both plasmids were treated with a range of concentrations of doxycycline for 48 h. Culture medium was then isolated and assessed for ChABC activity via colorimetric (spectrophotometric) assay. Without doxycycline administration, medium from co-transfected cells had some low basal ChABC activity (14% ± 4.5 U/ml), which was not increased by 1 ng/ml doxycycline administration. In contrast, application of 10 ng/ml doxycycline initiated the induction of *ChABC* gene expression (43% ± 4.4 U/ml), relative to maximal activity achieved by application of 100 ng/ml doxycycline (87% ± 7.6 U/ml) (Fig. 2B). Thus, *in vitro* co-transfection of dox-i-ChABC plasmids confer dox-sensitive expression of active ChABC enzyme.

### Doxycycline administration induces expression and secretion of active ChABC *in vivo*

We then sought to assess whether the dox-i-ChABC gene therapy system could be used to deliver ChABC, *in vivo*, to the injured spinal cord. LV.PGK.GARrtTA and LV.TRE.ChABC (Fig. 2A) were combined in equal ratio and injected either side of a midline cervical contusion injury at spinal level C5/6 and ChABC expression was assessed after 2.5 weeks ± doxycycline. In the presence of doxycycline, expression of the *ChABC* gene was successfully induced, to a similar magnitude as that in the positive control (constitutive) LV.PGK.ChABC. LV.PGK.GARrtTA and LV.TRE.ChABC co-injection without doxycycline administration resulted in very low gene expression [*F*(3,7) = 27.13, *P = *0.0003, one-way ANOVA, Tukey’s *post hoc*] (Fig. 2C). Translation of the *ChABC* gene and secretion of active enzyme was confirmed by immunostaining for C-4-S, a non-reducing epitope only exposed following CS-GAG digestion by the ChABC enzyme. C-4-S immunoreactivity in sagittal spinal cord sections through the lesion epicentre revealed extensive matrix modification by dox-i-ChABC (Fig. 2D, left), of a comparable magnitude to that observed with LV.PGK.ChABC (Fig. 1D, right). The pattern of intense C-4-S expression extended to spinal levels rostral (C3) and caudal (C7) to the lesion. Little matrix digestion was observed in distal segments of the thoracic (T10) or lumbar (L3) spinal cord at this early (2.5 week) time point. A small amount of C-4-S immunoreactivity was apparent in the spinal cord of dox− rats (Fig. 2D, middle) in localized regions close to the injection sites, revealing a low-level of ‘leaky’ transgene expression. Thus, our dox-i-ChABC system enables doxycycline-sensitive expression of active ChABC enzyme *in vivo* within the injured mammalian spinal cord.

### Dox-i-ChABC treatment can be temporally regulated *in vivo*

**Figure 4:**
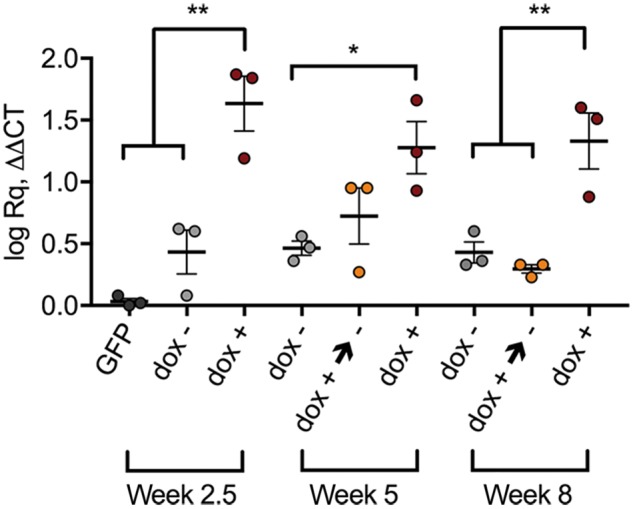
**Dox administration and removal allows temporal regulation of *ChABC* gene expression in the injured spinal cord.** *ChABC* gene expression as measured by qPCR on RNA extracted from spinal cord injury tissue epicentre and injection site following different periods of doxycycline (dox) exposure is regulated by doxycycline. Low levels of ‘leaky’ transgene expression are apparent at all time points following dox-i-ChABC vector injection without doxycycline administration (dox−, grey data points). At 2.5 weeks, high *ChABC* gene expression is achieved with doxycycline administration [*F*(2,6) = 25.67, *P = *0.0011, one-way ANOVA, Tukey’s *post hoc*]. At 5 weeks, following sustained administration of doxycycline (red data points), *ChABC* gene expression is maintained whereas removal of doxycycline at 2.5 weeks (yellow data points) results in lower transgene expression [*F*(2,6) = 5.204, *P = *0.0489, one-way ANOVA, Tukey’s *post hoc*]*.* By 8 weeks, ChABC expression has decreased to baseline levels following removal of doxycycline at 2.5 weeks (yellow data points), while high transgene levels are maintained with sustained doxycycline administration (red data points) [*F*(2,6) = 15.87, *P = *0.0040, one-way ANOVA, Tukey’s *post hoc*] (dox− versus dox+→−, *P = *0.7895; dox− versus dox+, *P = *0.0096; dox+→− versus dox+, *P = *0.0049).

Following confirmation that *ChABC* gene expression could be induced in the injured spinal cord in the presence of doxycycline, we then assessed whether gene expression could be regulated by manipulating the period of doxycycline exposure (validation of the on/off gene switch). QPCR was used to measure *ChABC* mRNA expression in spinal cord tissue at different post-injury time points after different doxycycline regimes (Figs 3 and 4) revealing differential *ChABC* gene expression [*F*(8,18) = 11.23, *P* < 0.001, one-way ANOVA, Tukey’s *post hoc*]. Relative to rats injected with LV.PGK.GFP (which do not express ChABC as it is a non-endogenous gene, and thus serve as negative controls), at 2.5 weeks following dox-i-ChABC vector injection with doxycycline administration ChABC expression was induced [*F*(2,6) = 25.67, *P = *0.0011, one-way ANOVA, Tukey’s *post hoc*]. Without doxycycline administration (dox−) there was a low level of ‘leaky’ transgene expression, and this low level transgene expression without doxycycline was apparent at all time points (Fig. 4). However, at 2.5 weeks the low level expression without doxycycline was significantly (17-fold) less than *ChABC* gene expression achieved with doxycycline administration (Fig. 4). At 5 weeks, following sustained administration of doxycycline, *ChABC* gene expression was maintained [*F*(2,6) = 5.204, *P = *0.0489, one-way ANOVA, Tukey’s *post hoc*], whereas removal of doxycycline at 2.5 weeks resulted in lower transgene expression (not significant at this time point, *P = *0.1598). By 8 weeks ChABC expression decreased to baseline levels following removal of doxycycline at 2.5 weeks, in contrast to sustained expression of *ChABC* mRNA with maintained doxycycline administration [*F*(2,6) = 15.87, *P = *0.0040, one-way ANOVA, Tukey’s *post hoc*] (Fig. 4). Thus, doxycycline administration and removal allows temporal regulation of *ChABC* gene delivery following experimental spinal cord injury. This confirms the functionality of the dox-i-ChABC on/off gene switch *in vivo* within the injured mammalian spinal cord.

### Dox-i-ChABC treatment promotes recovery of ladder walking performance following cervical contusion injury in adult rats

**Figure 5:**
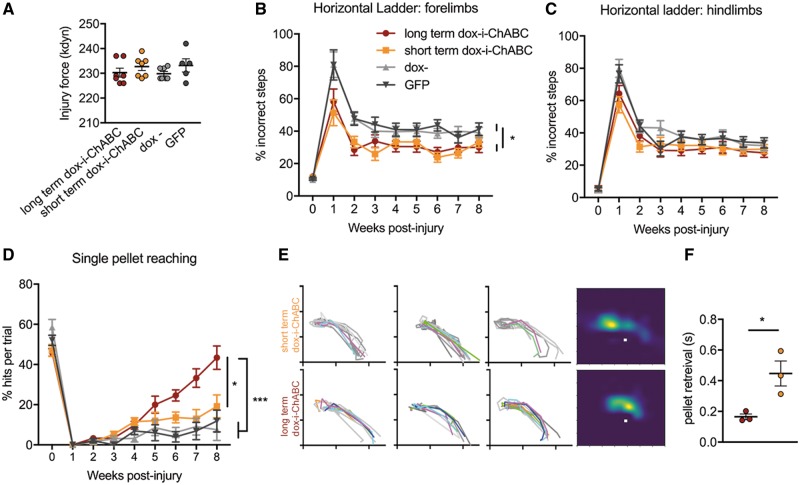
**Long- and short term dox-i-ChABC treatment promotes differential recovery following cervical contusion injury in adult rats.** (**A**) Contusion device impact force measurements did not differ between treatment groups, confirming consistent injuries across all groups [*F*(3,22) = 0.9582, *P = *0.4299, one-way ANOVA, *n = *7, 7, 7, 5). (**B**) Assessment of forelimb function using the horizontal ladder task revealed a significant improvement in performance following either long or short-term dox-i-ChABC treatment, relative to controls [*F*(3,21) = 6.875, *P = *0.02, two-way RM-ANCOVA, Bonferroni *post hoc*, *n = *7, 7, 7, 5]. (**C**) No significant differences were observed in hindlimb function on the horizontal ladder task [*F*(3,21) = 2.473, *P = *0.09, two-way RM-ANCOVA, Bonferroni *post hoc*, *n = *7, 7, 7, 5]. (**D**) Assessment of skilled reaching using the Whishaw window showed that while all groups were severely impaired in reaching and grasping ability at early post-injury time points, rats in the long-term dox-i-ChABC treatment group showed significant improvements in pellet reaching performance over time, with recovery emerging around 5 weeks post-injury and continuing throughout the testing period, with significantly improved performance in comparison to all other groups [*F*(3.17) = 9.643, *P = *0.001, two-way RM-ANCOVA, Bonferroni *post hoc*]. (**E**) Manual kinematic tracing of paw trajectory during assessment at the final 8 week time point. ‘Hits’ are coloured and successful pellet retrievals following more than one attempt are grey. Heat maps show paw location in space during the grasp phase relative to the sugar pellet (white squares). Long term dox-i-ChABC treated animals have a focused paw position in close proximity to the pellet during the grasping phase. Paw location of short-term dox-i-ChABC animals is less focused and frequently further from (beyond) the pellet, reflecting a repetitive grasping motion before successful retrieval. (**F**) The average time taken to successfully retrieve a pellet was significantly faster in long-term dox-i-ChABC animals compared to short term (*t*-test *P = *0.028, *n = *3, 3).

We next assessed the efficacy of dox-i-ChABC treatment in promoting functional recovery following cervical spinal contusion injury and whether long term versus short term treatment confers more benefit. Contusion device impact force measurements for each rat confirmed injuries were of equal severity across treatment groups (Fig. 5A). Assessment of forelimb function using the horizontal ladder, a task requiring sensorimotor integration, revealed improved performance following either dox-i-ChABC treatment for 2.5 or 8 weeks, relative to controls (Fig. 5B). While both dox− and GFP treated animals at 1 week post-injury made 81.0% ± 7.9 and 81.0% ± 9.3 footslips, respectively, those in dox-i-ChABC groups were already more accurate in their paw placement, making fewer slips (58.0% ± 7.8 and 51.3% ± 7.9 for long-term dox-i-ChABC and short-term dox-i-ChABC, respectively). This early effect was maintained into the chronic post-injury time points, where there was a significant effect of treatment over time. The long- and short-term dox-i-ChABC groups made significantly fewer incorrect steps than the dox− and GFP groups [*F*(3,21) = 6.875, *P = *0.02, two-way RM-ANCOVA, Bonferroni *post hoc*]. A trend for improvement with dox-i-ChABC treatment was observed for hindlimb performance, although this was not statistically significant [*F*(3,21) = 2.473, *P = *0.09, two-way RM-ANCOVA] (Fig. 5C). Thus, short-term dox-i-ChABC treatment appears sufficient to induce early improvements in ladder walking performance, with no greater improvement conferred by longer term dox-i-ChABC.

### Sustained dox-i-ChABC treatment promotes recovery of skilled reaching following cervical contusion injury

We next assessed whether dox-i-ChABC could promote recovery in a measure of skilled reaching and grasping. Following pre-training, rats in all groups made a similar number of ‘hits’ per ‘trial’ (successfully acquired pellets in a single reaching movement) (Whishaw *et al.*, 2008), which dropped to zero in all groups one week following injury. However, rats in the long-term dox-i-ChABC treatment group continued to improve over time, with improvements over all other treatment groups emerging around 4 weeks post-injury and continuing thereafter [*F*(3.17) = 9.643, *P = *0.001, two-way RM-ANCOVA, Bonferroni *post hoc*; Fig. 5D]. Pairwise comparisons collapsed across all time points revealed not only a significant recovery in long-term dox-i-ChABC treated rats relative to dox− and GFP controls (*P = *0.002, *P = *0.001, respectively), but also significantly greater improvement than the short-term dox-i-ChABC treatment group (*P = *0.033). Scores at Week 8 were 43.4% ± 5.8, 19.2% ± 5.7, 7.5% ± 5.3 and 11.9% ± 5.4 for long-term dox-i-ChABC, short-term dox-i-ChABC, dox− and GFP, respectively (Fig. 5D and Supplementary Videos 1–4).

To investigate this effect further, a representative sample of animals from the two dox-i-ChABC groups were analysed at the final 8-week time point using manual kinematic tracing. Reaches that led to successful pellet retrieval were traced based on the position of the middle proximal phalange of the paw. Figure 5E shows reaching and grasping traces from each rat, where first time ‘hits’ are coloured and successful pellet retrievals following more than one attempt (‘pellets’; Whishaw *et al.*, 2008) are grey (i.e. more colour signifies greater skill and precision). This highlights that long-term dox-i-ChABC-treated animals more accurately retrieved the pellet in fewer attempts. From these traces, information as to paw location in space, during the grasping phase, can be represented as a heat map for each group, relative to the sugar pellet (white square). Long term dox-i-ChABC treated animals have a focused paw position in close proximity to the pellet during grasping phase (Fig. 5E, bottom heatmap). In contrast, the paw position of short-term dox-i-ChABC treated animals is spread and frequently further from (beyond) the pellet, reflecting repetitive grasping motion before successful retrieval (Fig. 5E, top heatmap). This represents an ongoing deficit in the pronation and grasping phase of reaching following short term treatment, whereas long-term dox-i-ChABC treated animals have recovered pronation, grasping, supination and ability to retrieve the pellet upon targeted reach. This is additionally reflected by the average time taken to successfully retrieve a pellet, where long-term dox-i-ChABC treated animals successfully retrieved the pellet almost three times faster than those in the short-term dox-i-ChABC group (0.16 ± 0.02 and 0.45 ± 0.08 s, respectively, *t*-test *P = *0.028) (Fig. 5F). Thus, only sustained dox-i-*ChABC* gene therapy (with doxycycline administered for the full 8 weeks) promotes recovery of skilled paw use and accurate grasping, indicating that long term CSPG digestion may be required for significant neuroplasticity of descending systems involved in skilled reaching.

### Dox-i-ChABC treatment improves conduction of ascending dorsal column sensory axons through the cervical contusion site

**Figure 6:**
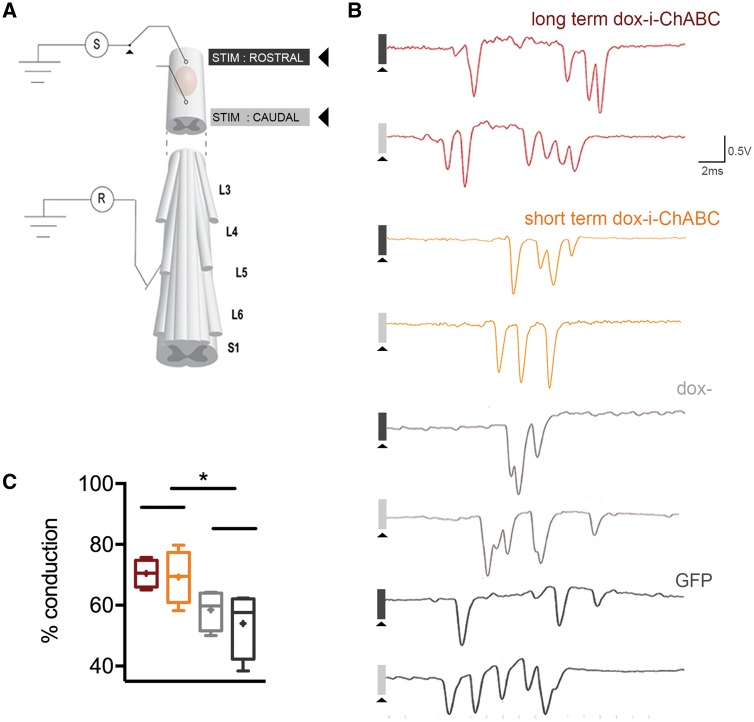
**Dox-i-ChABC treatment increases conduction of ascending dorsal column sensory axons through the cervical contusion site.** (**A**) Schematic of a terminal electrophysiological preparation to assess dorsal column sensory axon conduction. Dorsal column sensory axons are activated antidromically by a stimulus caudal to the lesion, and single unit action potentials recorded from teased fine-filaments of lumbar roots. The stimulus is then switched rostral to the injury and the percentage of action potentials still remaining from this sample quantified. (**B**) Representative example traces from each group show single unit action potentials which are quantified when stimulating caudal to the lesion and those remaining when stimulating rostral to the lesion (some units are present at similar latency in the traces but these are distinguishable during experimentation as they are sequentially recruited by incremental increases in stimulus amplitude). (**C**) Quantification of conduction shows that both short-term and long-term dox-i-ChABC treated groups had an increased percentage of axons able to conduct through the injury compared to dox− and GFP treated controls [*F*(3,12) = 3.961 one-way ANOVA *P = *0.036, Fisher’s LSD]. Boxplot whiskers are minimum to maximum with mean indicated as a plus symbol.

To assess physiological conduction properties of dorsal column sensory axons traversing the contusion injury site we used a terminal electrophysiological preparation, whereby single unit action potentials were recorded from teased fine-filaments of lumbar roots after antidromic stimulation rostral and caudal to the lesion (Fig. 6A). Following the final behavioural time point, at 8 weeks post-injury, both long-term and short-term dox-i-ChABC-treated groups showed a significant improvement of sensory fibre conduction, with an increased percentage of axons able to conduct through the injury (70.4% ± 2.3 and 69.3% ± 4.4, respectively), compared to dox− and GFP-treated controls (58% ± 3.3 and 54% ± 5.5, respectively) [*F*(3,12) = 3.961 one-way ANOVA *P = *0.036, Fisher’s LSD] (Fig. 6B and C). Thus, short-term dox-i-ChABC treatment is sufficient to confer increased conduction of dorsal column sensory afferents, with no greater improvement conferred by longer-term dox-i-ChABC treatment.

### Sustained dox-i-ChABC treatment is associated with increased density and distribution of vGlut1+ spinal innervation

**Figure 7:**
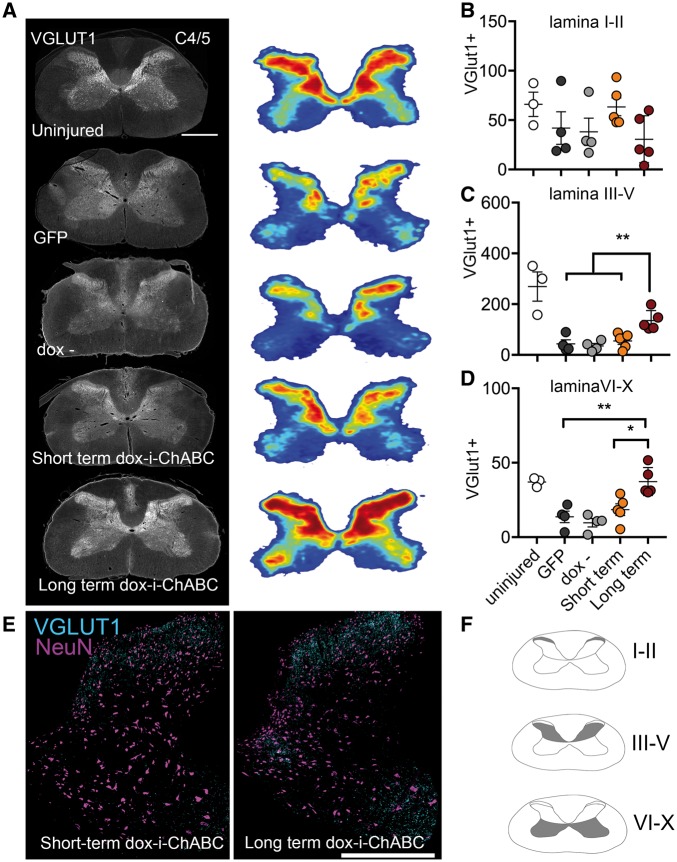
**Sustained dox-i-ChABC treatment is associated with increased density and distribution of vGlut1+ spinal innervation rostral to the lesion.** (**A**) VGlut1 immunoreactivity in representative transverse sections at spinal level C4/5 and threshold analysis of vGlut1 positive staining represented as density distribution heat maps reveals the normal vGlut1 innervation pattern in a naïve uninjured spinal cord and a marked decrease in vGlut1 immunoreactivity following C5/6 contusion injury in all groups except the long-term dox-i-ChABC treated group, where intense vGlut1 immunoreactivity is apparent throughout the spinal grey matter. (**B**) Quantification of vGlut1 immunopositive pixels per µm^2^ in laminas I–II revealed no differences between groups [*F*(3,14) = 1.452, *P = *0.2701, one-way ANOVA]. (**C**) VGlut1 immunoreactivity was significantly increased in the long-term dox-i-ChABC treatment group in lamina III–V, compared to all other injured groups [*F*(3,14) = 9.762, *P = *0.001 one-way ANOVA, Tukey’s *post hoc*]. (**D**) VGlut1 immunoreactivity was significantly increased in the long-term dox-i-ChABC treatment group in lamina VI–X compared to all other injured groups [*F*(3,14) = 10.47, *P = *0.0007 one-way ANOVA, Tukey’s *post hoc*]. (**E**) Representative sections showing co-localization of vGlut1 with the neuronal marker NeuN shows greater vGlut1 expression in lamina III–X in the long-term dox-i-ChABC treatment group relative to the short-term dox-i-ChABC treatment group. For individual channels see Supplementary Fig. 1. Scale bar = 0.5 mm.

**Figure 8:**
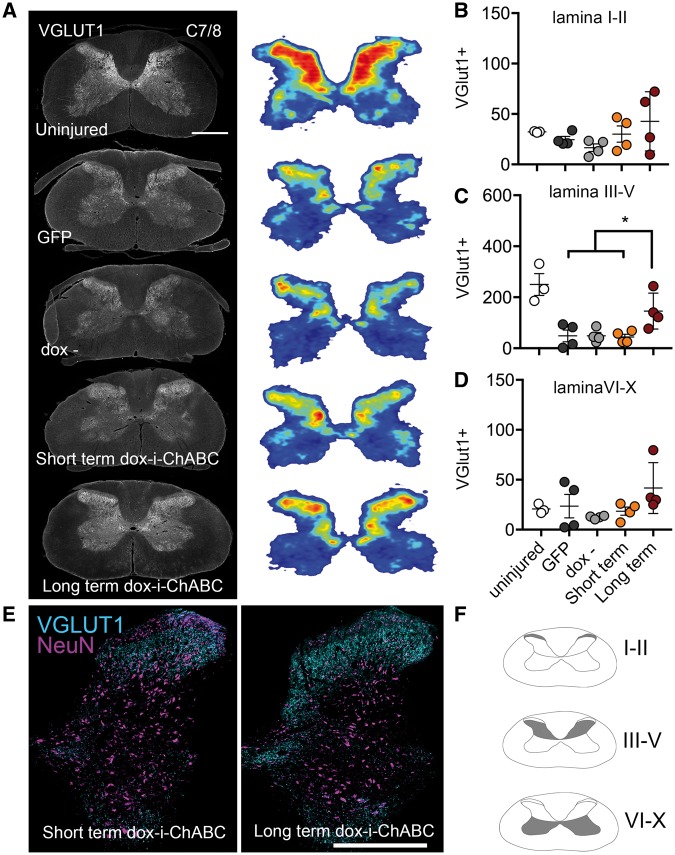
**Sustained dox-i-ChABC treatment is associated with increased density and distribution of vGlut1+ spinal innervation caudal to the lesion.** (**A**) VGlut1 immunoreactivity in representative transverse sections at spinal level C7/8 and threshold analysis of vGlut1+ staining represented as density distribution heat maps reveals the normal vGlut1 innervation pattern in a naïve uninjured spinal cord and a decrease in vGlut1 immunoreactivity following contusion injury. (**B**) Quantification of vGlut1 immunopositive pixels in laminas I–II revealed no differences between groups [*F*(3,12) = 1.993, *P* = 0.1688, one-way ANOVA]. (**C**) In lamina III–V the long-term dox-i-ChABC treated group had significantly higher vGlut1+ immunoreactivity compared to all other injured groups [*F*(3,12) = 4.715, *P = *0.0213, one-way ANOVA, Tukey’s *post hoc*]. (**D**) No significant differences between groups were observed in vGlut1 expression in lamina VI-X [*F*(3,12) = 1.629, *P = *0.2347, one-way ANOVA]. (**E**) Representative sections showing co-localization of vGlut1 with the neuronal marker NeuN shows greater vGlut1 expression in lamina III–V in the long-term dox-i-ChABC treatment group relative to the short-term dox-i-ChABC treatment group. Scale bar = 0.5 mm.

Following spinal cord injury, descending excitatory input is severely disrupted. Since ChABC treatment has previously been shown to promote neuroplasticity (Barritt *et al.*, 2006; Alilain *et al.*, 2011; Starkey *et al.*, 2012), we investigated whether dox-i-ChABC treatment could alter the distribution and density of the presynaptic excitatory marker vGlut1 in the spinal cord. Threshold analysis for vGlut1+ boutons, and heat maps representing their distribution in grey matter at spinal levels C4/5 rostral to injury, revealed that following injury there is a decrease and change in spatial distribution of excitatory vGlut1+ fibres relative to control uninjured spinal cord tissue (Fig. 7A). Regional analysis to quantify vGlut1 expression in different spinal lamina revealed a similar pattern of vGlut1 expression in superficial lamina (I-II) in all animals, with no significant differences observed between groups [*F*(3,14) = 1.452, *P = *0.2701, one-way ANOVA; Fig 7B]. However, in deeper lamina regions, lamina III–V (Fig. 7C) and lamina VI–X (Fig. 7D) rats receiving sustained dox-i-ChABC for the 8-week duration of the study showed significantly increased immunoreactivity for vGlut1, compared to all other injury and treatment groups [*F*(3,14) = 9.762, *P = *0.001 one-way ANOVA, Tukey’s *post hoc* and *F*(3,14) = 10.47, *P = *0.0007 one-way ANOVA, Tukey’s *post hoc*, respectively] with a distribution pattern within grey matter similar to that found in the uninjured spinal cord. Further examination of vGlut1 expression in sections co-stained with NeuN revealed intense vGlut1 expression distributed throughout lamina III–X in the long-term dox-i-ChABC treated group, compared to markedly lower levels and distribution of vGlut1 in short-term dox-i-ChABC group (Fig. 7E and Supplementary Fig. 1). VGlut1 expression was also examined at spinal levels C7/8 caudal to the injury (Fig. 8) and again vGlut1 threshold analysis revealed a decrease in distribution of excitatory vGlut1+ fibres following injury, relative to control uninjured spinal cord tissue (Fig. 8A). Regional analysis revealed no significant differences in vGlut1 expression between groups in superficial lamina [*F*(3,12) = 1.629, *P = *0.2347, one-way ANOVA; Fig 8B], but significantly increased vGlut1 immunoreactivity in the long-term dox-i-ChABC treated group compared to all other injury and treatment groups in lamina III-V [*F*(3,12) = 4.715, *P = *0.0213, one-way ANOVA, Tukey’s *post hoc*; Fig. 8C, E and Supplementary Fig. 1]. No significant differences between groups were observed in vGlut1 expression in lamina VI–X [*F*(3,12) = 1.629, *P = *0.2347, one-way ANOVA; Fig. 8D]. Hence, only long-term dox-i-ChABC led to increased density of vGlut1+ innervation rostral and caudal to the injury, indicating that long term CSPG digestion promotes enhanced innervation by descending motor pathways. Finally, we assessed serotonergic innervation caudal to the injury, at level T2, and observed increased density of serotonergic fibre projections in the intermediolateral columns and the ventral horn in both short term and long-term dox-i-ChABC treated animals, compared to GFP and dox− controls [*F*(3,8) = 7.807, *P = *0.0092 for intermediolateral columns and *F*(3,8) = 7.169, *P = *0.0118 for ventral horn, one way ANOVA, Tukey’s *post hoc*; Supplementary Fig. 2].

## Discussion

Here we demonstrate a novel immune-evasive gene switch that enables regulated delivery of ChABC in the injured mammalian spinal cord. This provides an experimental tool to control delivery (by effectively switching the gene on and off) and understand the role of timing in ChABC treatment, as well as a step towards creating a clinically applicable viral vector system. Dox-i-ChABC effectively mediates temporal control over CS-GAG removal, where administration of doxycycline, a safe and widely used pharmacological agent, induces high expression of the *ChABC* gene and enables secretion of the active enzyme and extensive matrix digestion. Removal of doxycycline returns *ChABC* gene expression to baseline levels, with some low ‘leaky’ transgene expression. We use this to reveal that short-term *ChABC* gene therapy is sufficient to replicate prior reported functional improvements and find that long term administration elicits additional recovery of skilled forelimb reaching and grasping behaviours and this is associated with vGlut1+ fibre remodelling in the spinal cord.

### Regulating *ChABC* gene delivery

This is the first demonstration of a regulated chondroitinase gene therapy system *in vivo.* One previous attempt to generate Tet-On chondroitinase, using an adenoviral vector encoding chondroitinase AC has been reported and used in a human astrocytoma cell line (Curinga *et al.*, 2007) but this was not tested *in vivo.* Here, we use a tetracycline derivative to induce expression of an optimized *ChABC* transgene in order to exert temporal control over enzyme delivery *in vivo*, in the mammalian spinal cord, following a clinically-relevant cervical level spinal contusion injury to adult rats. The ability to precisely control transgene expression is important, both for extending the range of biological applications of viral vector delivery and for advancing clinical development. An ideal system would be able to stably maintain expression of a particular gene within the desired therapeutic range but also allow gene expression to be turned off to avoid potential side effects associated with long term delivery of some genes (Stieger *et al.*, 2009). For example, off target side effects such as aberrant sprouting and weight loss have been reported with long term viral vector delivery of glial cell line-derived neurotrophic factor (GDNF) (Georgievska *et al.*, 2002; Manfredsson *et al.*, 2009; Su *et al.*, 2009) and may limit its effectiveness for application to Parkinson’s disease and other disorders, leading to efforts to develop inducible GDNF vector systems (Chtarto *et al.*, 2016). Long-term viral delivery of some therapeutic transgenes to the injured spinal cord has also been reported to induce deleterious effects. For example, BDNF delivered via AAV intraspinal injection to cervical hemisected rats was shown to enhance axonal growth, but was also associated with hyperreflexia and spasticity, which was attributed to excitatory effects of chronic BDNF over expression (Lu *et al.*, 2012; Fouad *et al.*, 2013). Neuroplasticity effects of ChABC have been widely demonstrated in numerous preclinical studies and related to restored connectivity and recovery of many functions (Pizzorusso *et al.*, 2002; Garcia-Alias *et al.*, 2009; Alilain *et al.*, 2011; Wiersma *et al.*, 2017). However, there is the possibility that long-term ChABC overexpression could eventually lead to maladaptive remodelling. Although no negative effects have been documented thus far following ChABC delivery to the injured or uninjured spinal cord, for example with measures of pain thresholds showing no enhanced sensitivity (Barritt *et al.*, 2006; Galtrey *et al.*, 2007; Karimi-Abdolrezaee *et al.*, 2010), even with sustained *ChABC* gene therapy for up to 12 weeks (Bartus *et al.*, 2014). Nevertheless, clinical translation of ChABC will likely require a safeguard against potential aberrant plasticity.

In addition to improving clinical acceptability, the ability to precisely modulate the period of ChABC administration opens possibilities for exploring windows of neuroplasticity, which could be particularly potent if combined with rehabilitation paradigms. The neuroplasticity afforded by unmodified bacterial ChABC enzyme treatment administered early post-injury has previously been shown to open a window during which rehabilitation could promote upper limb recovery, leading to improved manual dexterity in skilled reaching tasks in dorsal hemisected rats (Garcia-Alias *et al.*, 2009). Controlled delivery with dox-i-ChABC offers the opportunity to establish whether late stage windows of neuroplasticity are possible in the chronically injured spinal cord, as has recently been shown in experimental models of chronic stroke where bacterial ChABC enzyme injections dramatically potentiated the efficacy of rehabilitative training (Wiersma *et al.*, 2017), or indeed whether multiple windows of neuroplasticity are possible. Timing of intervention and training is also an important issue that can be explored with dox-i-ChABC, as for other neuroplasticity-inducing interventions such as anti-Nogo immunotherapy where sequential (rather than concurrent) timing of the neuroplasticity intervention and the rehabilitation protocol was shown to improve recovery from experimental stroke and spinal cord injury (Wahl *et al.*, 2014; Chen *et al.*, 2017). Furthermore, it is likely that rehabilitation paradigms, which aim to consolidate and strengthen correct connectivity, may benefit from restoring appropriate neural stability following training by switching off ChABC.

Thus, the ability to regulate *ChABC* transgene expression represents a significant advance in exerting greater control over neuroplasticity. The ability to modulate ChABC delivery could also have a range of applications other than in the treatment of spinal cord injury. It may represent an experimental tool in studies of developmental neurobiology, learning and memory whereby ChABC-mediated degradation of CSPG-rich perineuronal nets is known to effectively reopen the critical period and re-enable induction of ocular dominance plasticity and the erasure of subsequently acquired fear memories (Pizzorusso *et al.*, 2002; Gogolla *et al.*, 2009). Temporary or repeated induction of large-scale plasticity, or greater control over critical period elongation could be achievable by using dox-i-ChABC in these systems. Regulated ChABC delivery also has potential wider applications for other preclinical models of CNS disorder and trauma. For example, ChABC bacterial enzyme injection has been applied to experimental stroke models, with observed increases in midline sprouting of corticospinal tract axons and recovery of forelimb sensorimotor function (Soleman *et al.*, 2012) and tau pathology models, where restoration of memory was transiently restored (Yang *et al.*, 2015). These effects could potentially be enhanced with longer term delivery, or with multiple treatment windows, using dox-i-ChABC. Furthermore, the wider implications of developing an effective immune-evasive method of regulating gene expression in the mammalian CNS may be expanded to other neurological disorders with an autoimmune or inflammatory component such as multiple sclerosis or amyotrophic lateral sclerosis.

### Tet-regulated gene therapy in the injured spinal cord

The current study represents the first use of an immune-stealth gene therapy vector system to deliver a transgene to the injured rodent spinal cord. It has been questioned whether the ‘immune-privileged’ CNS could evade the issue of rtTA recognition and removal of transduced cells (Chtarto *et al.*, 2016), observed following gene delivery to the peripheral nervous system in rats (Markusic *et al.*, 2010) and primates (Favre *et al.*, 2002; Latta-Mahieu *et al.*, 2002). However, injury to the spinal cord results in non-resolving trauma-induced autoimmunity (Popovich *et al.*, 1996; Fleming *et al.*, 2006; Ankeny and Popovich, 2009; Pruss *et al.*, 2011). Thus, the potential for immune-mediated removal of transduced cells in the aggressive inflammatory environment of the injured spinal cord is likely to pose a problem for advancing Tet-regulated gene therapy to the injured CNS. Following lesion to basal forebrain cholinergic neurons, previous work has shown that Tet-Off delivery of NGF can be achieved via *ex vivo* transduction and transplantation of fibroblasts (Blesch *et al.*, 2001) or lentivirus injection, for 2 weeks (Blesch *et al.*, 2005). In the injured spinal cord, a further study demonstrated 6 weeks of neurotrophin delivery via transplantation of fibroblasts that were *ex vivo* transduced to express Tet-On BDNF (Blesch and Tuszynski, 2007) and BDNF delivery up to 8 weeks following *in vivo* transduction distal to a lateral hemisection injury and cellular transplantation site (Liu *et al.*, 2017). However, gene therapy treatments for spinal cord injury using *in vivo* transduction via intraparenchymal vector delivery in close proximity to a contusion site, where the environment is one of aggressive inflammation, is more likely to expose rtTA to antigen presenting cells. Indeed, here we examined whether the addition of a ‘stealth’ gene switch, which uses a chimeric transactivator fused with GAR, could confer immune-evasive properties following *in vivo* injection into the contused rat spinal cord. Following intraspinal injection and 2.5 weeks of doxycycline administration to activate gene expression, we found no interferon response and no enhanced CD8b response with the GARrTA dox-inducible ChABC vector system, in contrast to the classical rtTA system. This provides evidence that classical rtTA systems can elicit and/or exacerbate a T cell mediated immune response when injected into an immune compromised spinal cord injury environment, and that the stealth component of dox-i-ChABC can evade T cell recognition, at least at this post-injury time point. Furthermore, this is supported by recent work showing that even in the uninjured spinal cord the classical rtTA system is compromised over time (de Winter *et al.*, in preparation). A side by side comparison of rtTA and GARrtTA inducible vectors driving luciferase gene expression (enabling long term bioluminescence imaging) showed that rtTA-mediated gene expression undergoes shutdown upon repeated induction in rats, whereas GARrtTA-mediated gene expression is maintained; rtTA shutdown was associated with cell and tissue damage, presumably due to immune cell activation (de Winter *et al.*, in preparation). These data, together with the current findings, support previous *in vitro* findings, which used a bioassay for human antigen presentation to demonstrate that GAR protects rtTA from human cytotoxic T cell mediated recognition, confirming that the chimeric transactivator GARrtTA had an immune-evasive advantage over classical rtTA (Hoyng *et al.*, 2014) and support the need for addition of an immune evasive component for application of inducible vector systems to spinal cord injury. The present demonstration that dox-i-*ChABC* gene therapy both replicates and adds to our previous reported functional improvements with a non-regulatable ChABC vector (Bartus *et al.*, 2014; James *et al.*, 2015) together with the demonstration that a vector system designed to evade the immune system (Zaldumbide *et al.*, 2010; Hoyng *et al.*, 2014) has immune-stealth properties *in vivo* in the injured mammalian spinal cord represents an important experimental advance in the use of regulated gene therapy systems for spinal cord injury as well as a step towards increasing clinical feasibility of *ChABC* gene therapy.

### Dox-i-ChABC has low-level basal ‘leaky’ transgene expression

Some *ChABC* gene expression and enzyme activity was detected without doxycycline administration. Although these low levels of leaky gene expression did not appear to have any biological effects, at least in terms of affecting forelimb function and anatomical plasticity, it nevertheless would be an advantage to eliminate residual leakiness from the system. Although addition of GAR significantly improves upon traditional rtTA systems in terms of reduction in basal expression (Hoyng *et al.*, 2014), it may prove beneficial to further optimize the construct, using minimal activation domains such as those found through screening assays (Urlinger *et al.*, 2000) or viral evolution (Zhou *et al.*, 2006). Furthermore, the TRE tetO sequences are also amenable to modification to reduce background expression and increase sensitivity (Loew *et al.*, 2010; Roney *et al.*, 2016). Current efforts are ongoing to introduce these elements to enhance the robustness and translational potential of the dox-i-ChABC system. In addition, because AAV-based gene therapy has a well-established clinical safety profile, efficacy of dox-i-ChABC within an AAV delivery platform will be assessed. AAV-based gene therapy has been approved for clinical use in the treatment of genetic lipoprotein lipase deficiency (Gaudet *et al.*, 2012) and in the CNS, AAVs are the most commonly used vector in clinical trials targeting tumours and neuronal disorders (Choudhury *et al.*, 2017). However, recent success with lentiviral-mediated gene therapy for the treatment of cerebral adrenoleukodystrophy in children (Eichler *et al.*, 2017) also highlights the ongoing potential of lentivirus-based systems for clinical use.

### Temporal and task dependence of functional recovery

Here we found that both short term and long term ChABC delivery with dox-i-ChABC promoted improved performance on the horizontal ladder task as well as enhanced conduction of the ascending dorsal column projections. These functions have previously been found to be highly correlated (James *et al.*, 2011) and both were shown to be enhanced with our previous (non-regulated) *ChABC* gene therapy system (Bartus *et al.*, 2014; James *et al.*, 2015), where early improvements on the ladder were associated with neuroprotection due to modulation of the inflammatory response by large scale CSPG digestion (Bartus *et al.*, 2014). The finding that dox-i-ChABC treatment improved both ladder performance and ascending neurotransmission is an important confirmation that the new regulatable system confers the same functional benefits as our previous non-regulated vector. Furthermore, these data have provided important information on timing, since it appears that short term (2.5 weeks) treatment is sufficient to confer neuroprotective effects that are comparable to those observed with a non-regulated ChABC vector. Finally, dox-i-ChABC also led to an increase in descending serotonergic projections, again confirming previous observations, both with bacterial ChABC enzyme (Barritt *et al.*, 2006; Karimi-Abdolrezaee *et al.*, 2010; Alilain *et al.*, 2011) and with non-regulated *ChABC* gene therapy (Bartus *et al.*, 2014).

In addition to replicating previous findings with a non-regulated ChABC vector, we also wanted to assess efficacy of dox-i-ChABC in a task which requires skilled reaching and grasping performance and to determine the effect of timing on these functions. It appears that short term ChABC delivery is not sufficient to elicit recovery of skilled motor performance, since only long-term dox-i-ChABC treatment promoted significant recovery of skilled grasping ability. The late stage emergence of recovery on this task (improvements only began to emerge after 4 weeks) indicates that recovery of skilled hand function was not attributable to neuroprotection, but more likely to be due to neuroplasticity as a result of long term CS-GAG digestion. Accordingly, these animals also had a greater density of vGlut1+ innervation in the spinal cord rostral and caudal to the injury. VGlut1+ populations of neurons in the spinal cord constitute proprioceptive afferents synapsing on motor neurons (Rotterman *et al.*, 2014), corticospinal tract terminals (Du Beau *et al.*, 2012) and primary afferent fibres (Alvarez *et al.*, 2004). Feasibly, plasticity in any or all of these populations could contribute to the observed recovery, but density heat maps and regional specific analysis of spinal cord lamina (split into lamina I–II, III–V and IV–X) to delineate the spatial profile of plasticity showed increased immunoreactivity particularly in regions III–IV both above and below the injury, areas likely to represent grey matter innervation by the corticospinal tract. As the corticospinal projection is associated with rodent success in the Whishaw window reaching task (Whishaw *et al.*, 1993), it is possible that long-term treatment enables reorganization and compensatory connectivity of this descending pathway. Neuroplasticity of other descending systems may also be involved, for example dox-i-ChABC may augment the natural capacity of reticulospinal tract fibres derived from the nucleus reticularis gigantocellularis to form compensatory detour circuits with propriospinal neurons (Filli *et al.*, 2014). Future studies should characterize this further, with anatomical tract tracing of specific spinal pathways since the corticospinal tract, or pathways that can compensate for loss of its function, are of particular importance to human motor control and recovery of these descending systems are thought to be important determinants of independence following cervical spinal cord injury (Wirth *et al.*, 2008). Electrophysiological assessments of descending transmission should also be evaluated in the future, to evaluate conduction and connectivity in descending motor pathways involved in skilled reaching performance.

Thus, our novel immune-evasive gene switch represents a powerful experimental tool to temporally explore the effects of ChABC therapeutic treatment and an encouraging step towards generating a more clinically-feasible *ChABC* gene therapy strategy. Its use reveals temporally dependent, task-specific, functional effects where short term administration is sufficient to enable recovery of sensorimotor integration during walking and long term administration confers additional benefit to skilled reaching and grasping after cervical contusion injury. This preclinical study represents a significant advance since recovery of hand function is the highest rated priority for improving functional outcome in individuals with tetraplegia.

## Funding

This work was supported by the United Kingdom Medical Research Council (Senior Non-Clinical Fellowship Award G1002055 to E.J.B.), the International Spinal Research Trust and EndParalysis (TRI004_01 to E.J.B.; TRI004_02 to E.M.M; TRI004_03 to J.V.) and Wings for Life Spinal Cord Research Foundation (WFL-NL-17/16 to J.V.).

## Supplementary material

Supplementary material is available at *Brain* online.

## Supplementary Material

Supplementary Data

## Abbreviations

ChABC: chondroitinase ABC
CS-GAG: chondroitin sulfate glycosaminoglycan
CSPG: chondroitin sulfate protegoglycan
dox-i-ChABC: doxycycline inducible ChABC
GARrtTA: glycine alanine repeat fused to rtTA
PGK: phosphoglycerokinase
rtTA: reverse tetracycline controlled transactivator; TRE = tetracycline response element
