# Supplementary material for: Immune-evasive gene switch enables regulated delivery of chondroitinase after spinal cord injury
Source: Brain. 2018 Jun 14;141(8):2362–81. doi: 10.1093/brain/awy158 (PMC6061881; doi:10.1093/brain/awy158)
Supplement: Supplementary Data [file awy158_supp.zip › awy158-suppl_data/brain-2017-02164-File010.pdf]

Supplementary Figure 1 – Sustained dox-i-ChABC treatment is associated with increased density and distribution of vGlut1+ spinal innervation rostral and caudal to the lesion

Single and merged channels showing immunostaining for VGlut1 (cyan) and NeuN (magenta). Rostral to the lesion there is increased density of VGlut1+ expression in laminas III-X in the spinal cord of animals in the long-term dox-i-ChABC treatment group versus the short-term dox-i-ChABC treatment group. Caudal to the lesion, long-term dox-i-ChABC treatment results in increased density of vGlut1+ immunostaining particularly in laminas III-V.

Supplementary Figure 1

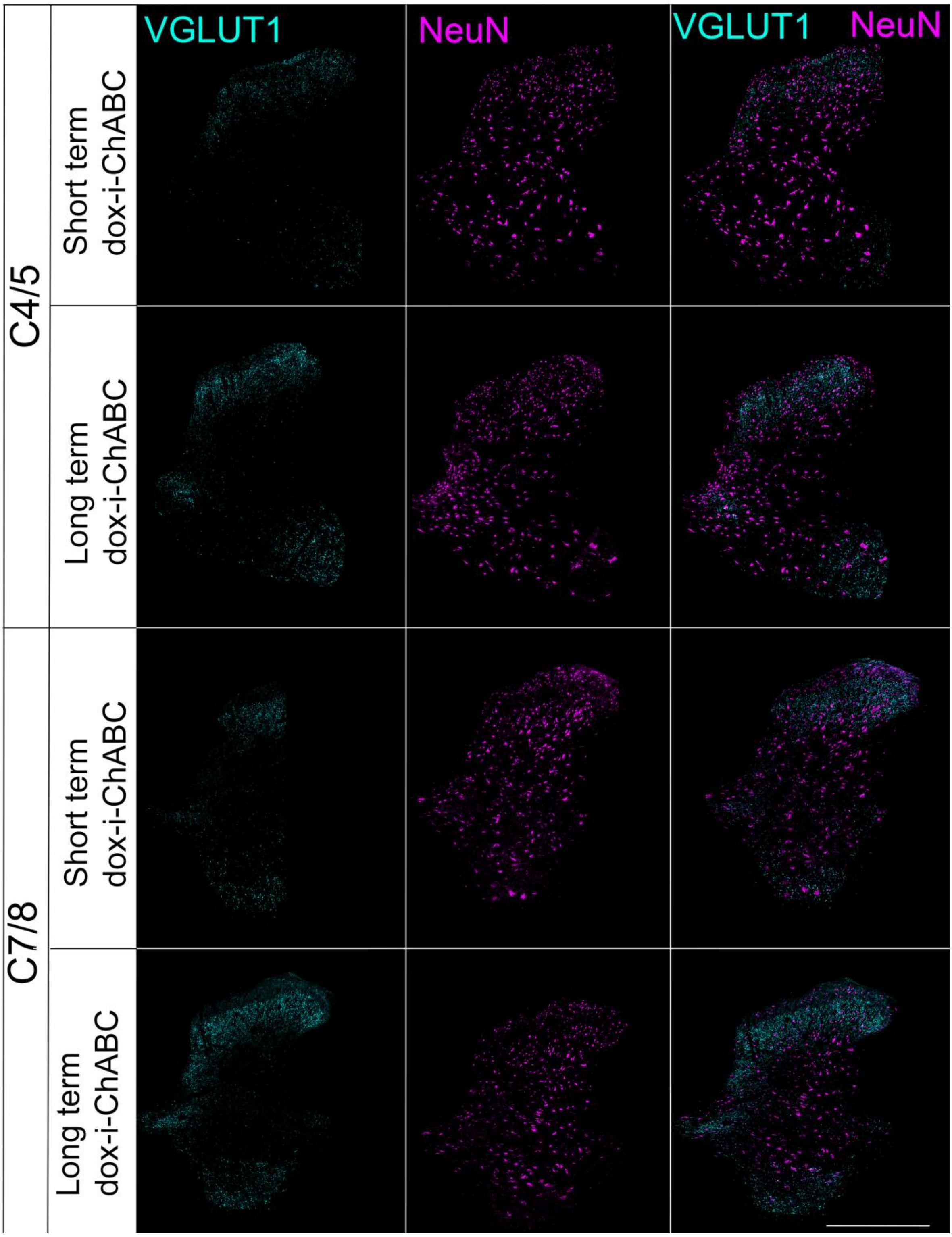

Supplementary figure 2

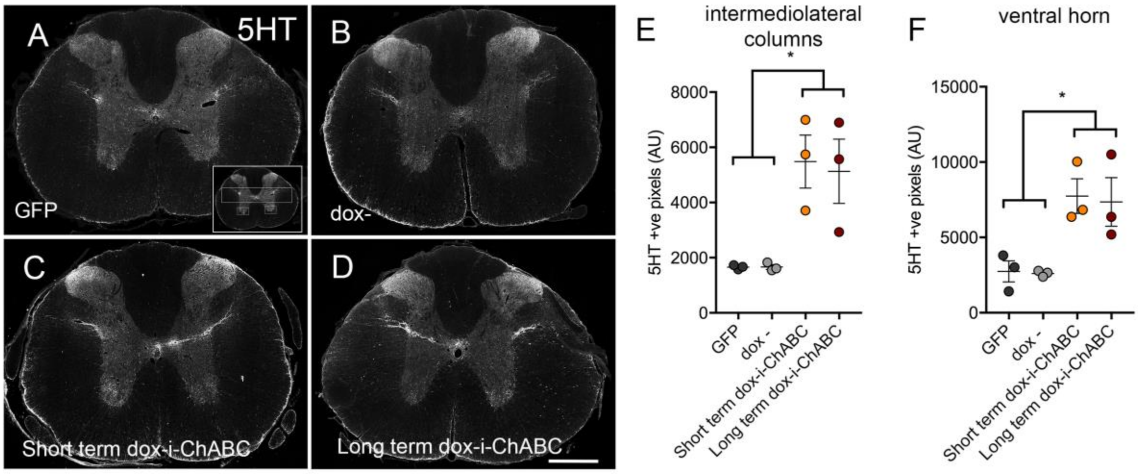

Supplementary Figure 2 – Both short and long-term dox-i-ChABC treatment leads to increased serotoninergic sprouting caudal to the lesion

Immunostaining for 5HT in transverse sections caudal to the injury at spinal level T2 in animals treated with (**A**) GFP, (**B**) dox-, (**C**) short-term dox-i-ChABC and (**D**) long-term dox-i-ChABC. Increased density of serotonergic fibres was observed in both short term and long-term dox-i-ChABC treated groups compared to controls, apparent in (**E**) the intermediolateral columns ($F_{3,8}$ = 7.807, P = 0.0092 one way ANOVA, Tukey's post hoc) and (**F**) the ventral horn ($F_{3,8}$ = 7.169, P = 0.0118 one way ANOVA, Tukey's post hoc).

Supplementary video 1 – Whishaw window reaching and grasping task: baseline

Rats were trained to reach for sucrose pellets with their dominant paw, through a window in a plexiglass box. Pellets were placed in an indented well, retrieval from which requires grasping and paw supination rather than dragging of the pellet towards the mouth. For inclusion in assessment all rats achieved >65% pellet retrieval at baseline.

Supplementary video 2 – Whishaw window reaching and grasping task: 8 weeks, doxycycline -.

At week 8 control rats reach for pellets, but do so inaccurately, displacing many and making multiple attempts before successful retrieval.

Supplementary Video 3 – Whishaw window reaching and grasping task: 8 weeks, short-term dox-i-ChABC treatment

At week 8, rats in the short-term dox-i-ChABC treatment group successfully reach for pellets but make inaccurate attempts to do so, frequently grasping without retrieving the pellet and taking multiple attempts before a successful retrieval.

Supplementary Video 4 – Whishaw window reaching and grasping task: 8 weeks, long-term dox-i-ChABC treatment

At week 8, rats in the long-term dox-i-ChABC treatment group more accurately retrieve sugar pellets at first-attempt (‘hits’) and rapidly acquire the pellet successfully.
